# Enhancing Landmark Point Detection in *Eriocheir Sinensis* Carapace with Differentiable End-to-End Networks

**DOI:** 10.3390/ani15060836

**Published:** 2025-03-14

**Authors:** Chong Wu, Shuxian Wang, Shengmao Zhang, Hanfeng Zheng, Wei Wang, Shenglong Yang

**Affiliations:** 1 East China Sea Fisheries Research Institute, Chinese Academy of Fishery Sciences, Shanghai 200090, China; cw825903048@163.com (C.W.); wangshx59@mail2.sysu.edu.cn (S.W.); zhenghf@ecsf.ac.cn (H.Z.); wangw@ecsf.ac.cn (W.W.); ysl6782195@126.com (S.Y.); 2 College of Information Technology, Shanghai Ocean University, Shanghai 201306, China; 3 School of Information Engineering, Huzhou University, Huzhou 313000, China

**Keywords:** *Eriocheir sinensis*, Chinese mitten crab, landmark detection, Gaussian heatmaps, deep convolutional neural network, DSNT module, generalization ability, power consumption

## Abstract

This research proposes using a neural network to detect and identify the landmark points of the carapace of the Chinese mitten crab, with the aim of improving efficiency in observation, measurement, and statistics in breeding and sales. A 37-point localization framework was developed for the carapace, with the dataset augmented through random distortions, rotations, and occlusions to enhance generalization capability. Three types of convolutional neural network models were used to compare detection accuracy, generalization ability, and model power consumption, with different loss functions compared. The results showed that the Convolutional Neural Network (CNN) model based on the Differentiable Spatial to Numerical Transform (DSNT) module had the highest R^2^ value of 0.9906 on the test set, followed by the CNN model based on the Gaussian heatmap at 0.9846. The DSNT-based CNN model exhibited optimal computational efficiency, particularly in power consumption metrics. This research demonstrates that the CNN model based on the DSNT module has great potential in detecting landmark points for the Chinese mitten crab, reducing manual workload in breeding observation and quality inspection, and improving efficiency.

## Simple Summary

This study investigates three convolutional neural network (CNN) architectures to detect 37 key points on the carapace of the Chinese mitten crab. To enhance model generalization, the dataset was augmented with random distortions, rotations, and occlusions. The models were evaluated based on their detection accuracy, generalization capability, and power consumption. The results indicate that the network incorporating the DSNT module outperformed others in both accuracy and resource efficiency. These findings highlight the potential of the DSNT-based network to significantly enhance the efficiency and precision of quality assessment and monitoring in Chinese mitten crab aquaculture.

## 1. Introduction

Chinese mitten crab (*Eriocheir Sinensis*), a species of Arthropoda, Crustacea, Decapoda, and Reptilian suborder, is highly valued in the market for its unique taste. It was introduced in Germany from China in the early 20th century, and its population exploded in the 1920s and 1930s, rapidly spreading to many Nordic rivers and estuaries. Commercial shrimp trawlers south of the San Francisco Bay collected the first Chinese mitten crab off the West Coast in 1992, and since then, it has become a delicacy all over the world, with significant economic value and potential [1]. In China, Chinese mitten crabs populations are categorized into four distinct ecotypes—Yangtze River, Liaohe, Oujiang, and Yellow River—based on geographical habitat variations. To study the morphological attributes of different populations of Chinese mitten crabs, multiple fields of scholars have conducted extensive research on their morphology. Scholars have proposed control groups based on larval characters to circumvent the adaptation of adult crabs to different environments, and they have measured and observed the white pattern, length, and width of the carapaces, the shape of the genitals, etc. [2,3]. With the rapid development of video surveillance technology and deep learning, convolutional neural networks (CNN) have been applied to images of crabs, leading to innovative research. For instance, Cui et al. [4] used a convolutional neural network to classify the sex of Chinese mitten crabs with high accuracy, and S Cao et al. [5] proposed a low-illumination enhancement technology to improve the detection of underwater live crabs. Wang et al. [6] used a convolutional neural network to identify the joints of the Atlantic blue crab, which provided some technical support for the meat picking of intelligent machines. However, the degree of automation of crab identification, classification, and processing is still low. In particular, there is a lack of research on fine landmark detection on crab carapaces. Accurate landmark detection can assist in extracting various morphological parameters, such as carapace length, tooth width, abdomen width, and plastron width, significantly reducing the manual workload. Thus, this study proposes a neural network approach to detect and identify landmark points of the carapace of the Chinese mitten crab, which can enhance the efficiency of observation, measurement, and statistics in the breeding and sale of Chinese mitten crab.

Landmark detection represents a critical domain in computer vision, with diverse applications including target matching [7], tracking [8], 3D reconstruction [9], among others. Traditionally, corner points have been used as essential point features for landmark detection. Harris corner detection [10] and Scale-invariant feature transform (SIFT) feature detection [11] are the commonly used feature point detection algorithms based on conventional computer vision. Although traditional methods like Harris corner detection and SIFT remain viable for certain applications, their reliance on hand-crafted features limits adaptability to complex biological structures like crab carapaces, prompting this study’s focus on deep learning approaches. With the advancement of deep learning, the landmark detection method based on neural networks has become one of the mainstream methods. The neural network-based landmark detection methods are mainly divided into two categories: fully connected regression and Gaussian heat map regression. In the fully connected regression method, a fully connected layer is added at the end of the convolutional neural network to map the feature map to the coordinate points. For instance, Hou et al. [12] proposed a multi-channel convolutional neural network for face alignment using multiple CNNs to learn local features and fully connected layers to learn global features. This method achieves state-of-the-art results on the 300 Face in-the-Wild (300 W) dataset [13]. One of the significant advantages of the fully connected regression method is its extraordinary intuitiveness. The fully connected layer can directly connect global features to the coordinates of feature points. However, the fully connected regression method significantly impairs the spatial generalization ability of the network [14], as it is too dependent on the distribution of the training data, which is more likely to cause overfitting. The Gaussian heat map regression method is the primary method in the field of human pose estimation [15,16]. The feature map of the Gaussian heat map is large, and the spatial generalization ability is strong. Despite its advantages, the Gaussian heatmap method suffers from limitations including high memory consumption, slow training/inference speeds, and non-end-to-end gradient propagation. Nevertheless, both methods have their own strengths and weaknesses, and researchers continue to explore new techniques and algorithms to improve the accuracy, efficiency, and applicability of landmark detection in various computer vision applications. Recent studies have demonstrated that Differentiable Spatial-to-Numerical Transformation (DSNT) offers an innovative solution to these challenges. By computing coordinate expectations from probability distributions, DSNT achieves end-to-end mapping from heatmaps to numerical coordinates, preserving the spatial generalization advantages of Gaussian heatmaps while eliminating their high memory overhead [17]. To address the low recognition accuracy of knob gears in unattended substations, Qin et al. [18] proposed a three-stage detection framework based on YOLOv4 and Darknet53-DUC-DSNT. This approach pioneers the application of deep learning-based keypoint detection for knob positioning. Yang et al. [19] developed a hybrid Mamba-Transformer architecture named RT-DEMT, which incorporates the DSNT framework to map heatmaps to numerical coordinates in an end-to-end manner, enabling accurate localization of acupuncture points. The DSNT framework establishes a new paradigm for landmark detection, with applications rapidly expanding into interdisciplinary fields such as ecology and robotic navigation.

The scarcity of research on landmark detection on the Chinese mitten crab carapace presents an opportunity for further exploration and development of this field. Accurate detection of landmark points on the crab carapace can facilitate automated measurement and analysis of various morphological characteristics of crabs. This can significantly reduce the time and effort required for manual measurements and enable more efficient and accurate data collection. Moreover, landmark detection on crab carapaces can be used for image correction, carapace comparison, and population assessment. For example, in the case of crab image correction, landmark detection can help to align and standardize crab images for better comparison and analysis. In population assessments, landmark detection can assist in identifying and tracking individual crabs, which can be useful for ecological research and conservation efforts. Thus, the development of accurate and efficient landmark detection methods for crab carapaces has significant practical applications and research value. To address these requirements, this study proposes a DSNT-based CNN architecture for Chinese mitten crab carapace landmark detection. Compared with conventional CNN frameworks utilizing fully connected layers or Gaussian heatmap regression, our method achieves superior accuracy, computational efficiency, and reduced computational footprint. This advancement enables precise and resource-efficient keypoint localization on crab carapaces, effectively meeting practical implementation requirements in ecological monitoring and aquaculture management.

## 2. Materials and Methods

### 2.1. Data

The Chinese mitten crabs used in this study were collected from Hongze Lake, one of the main producing areas of Chinese mitten crabs for food in China. Hongze Lake is the fourth largest freshwater lake in China, located in the lower reaches of the Huai River in Jiangsu Province (33°06′–33°40′ N, 118°10′–118°52′ E). A total of 52 crabs were collected, and 40–50 images were taken for each crab. The images were acquired using a OnePlus 6T smartphone (The OnePlus 6T smartphone was produced by OnePlus Technology Co., Ltd., headquartered in Shenzhen, Guangdong Province, China) with a main camera and a sub-camera. The main camera has 16 million pixels, a Sony IMX519 sensor (Tokyo, Japan), a 1.22 m pixel size, and an f/1.7 large aperture, while the secondary camera has 20 million pixels, a Sony IMX376K sensor, a 1.0 m pixel size, and an f/1.7 large aperture. During the shooting process, the smartphone was placed directly above the crab carapaces, and the left and right or front and rear tilt angles were controlled within 10° to ensure that all landmark points were captured. All images were taken on the same day under natural lighting conditions from 9 a.m. to 6 p.m. The sampling plan involved 52 Chinese mitten crabs collected from Hongze Lake, with no specific categorization by sex, length, or width during collection, as the primary focus was to develop a generalizable landmark detection model rather than to analyze morphological differences across these attributes. While length and width variations could influence landmark positioning (e.g., larger crabs may exhibit more pronounced tooth features), the study mitigated this by normalizing images to a consistent 512 × 512 resolution and applying data augmentation (Section 2.2.1), ensuring model robustness across size variations. Sampling followed an opportunistic design to mitigate potential selection bias in subsequent analyses, conducted on a single day to minimize environmental variability. However, this approach may limit representativeness, as the sample reflects only Hongze Lake’s population at one time point, potentially excluding seasonal or inter-population diversity (e.g., Yangtze vs. Liaohe populations). Future studies should incorporate stratified sampling by sex, size, and geographic origin to enhance representativeness and assess the impact of these factors on detection accuracy.

### 2.2. Method

#### 2.2.1. Data Processing Method

**Figure 1:**
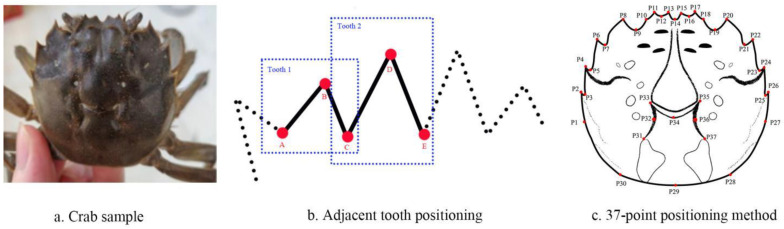
The positioning method of the carapace of Chinese mitten crab. Notes: In the figure, (**a**) provides a sample image of a crab, and (**b**) explains that each set of three landmarks forms a tooth, and neighboring teeth will share one landmark. In (**c**), the red dots denote the positions of the key points. Each point’s corresponding *p* value (P1, P2, etc.) denotes the numbering sequence of the points. A total of 37 key points are marked, including 27 key points at the tip of each tooth and the junction between adjacent teeth, as well as 10 key points for the rear edge and the M-shaped neck groove.

The perimeter of the carapace of Chinese mitten crabs is mainly composed of teeth, as shown in Figure 1a. The carapace has four frontal teeth on the front side, four lateral teeth on the left and right sides, and a relatively flat rear edge. The carapace also has a relatively apparent M-shaped neck groove in the middle. Based on these characteristics, the study proposes a method for locating 37 landmark points on the carapace of Chinese mitten crabs. Each tooth is positioned using three characteristic points: starting point, tooth peak, and end point for the twelve teeth (four frontal teeth, four left front teeth, and four right front teeth). Two adjacent teeth share a feature point, as shown in Figure 1b. For example, the C point is both the end point of Tooth 1 and the start point of Tooth 2. Thus, four frontal teeth can be located with nine feature points. Similarly, the left front teeth and the right front teeth are both located using nine feature points. The front, left, and right edges of the carapace are determined by 27 feature points, and the back edge is located using three landmark points. The M-shaped neck groove is located by seven landmark points. The complete 37 landmark points localization method is shown in Figure 1c. The annotation of feature points is conducted using the imgLab [20] open source tool (https://github.com/NaturalIntelligence/imglab, accessed on 1 March 2025).

**Figure 2:**
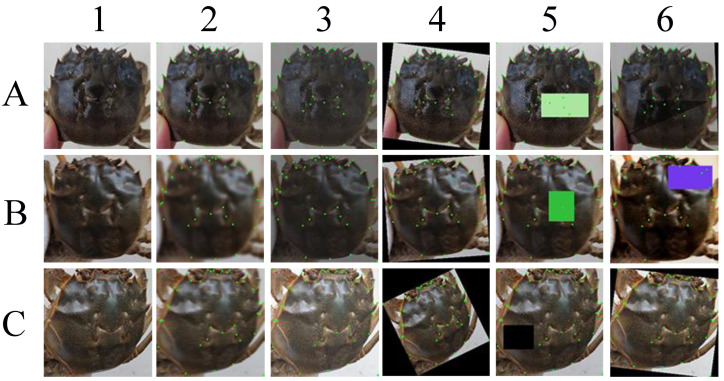
Methods of image augmentation.

The present study used a OnePlus 6T smartphone to capture images of the crabs, with 40–50 images taken for each crab. The shooting angles used were relatively uniform, and most obstacle occlusion problems were deliberately avoided during shooting. However, in practical applications, there may be many influencing factors, such as shooting equipment, shooting environment, shooting angle, and obstacles. Therefore, data augmentation is necessary for the study. As shown in Figure 2, the study used three groups of images to represent typical images, blurred images, and images with significant angular deviation. The images were subjected to various data augmentation methods, including random Gaussian blurring, random brightness and contrast processing, random rotation, and random occlusion, with a probability of 0.5 for each treatment. The processed images were marked with the processed landmark points, which is convenient for determining the accuracy of the landmarks. The final dataset for training was randomly combined with each data augmentation method using a probability of 0.5 to achieve a balanced effect. After data augmentation, the amount of image data reached 4600. To explore whether data augmentation can improve the model’s generalization ability, the original dataset and the augmented dataset were used in the base network to form a control group.

#### 2.2.2. Network Design Methods

**Figure 3:**
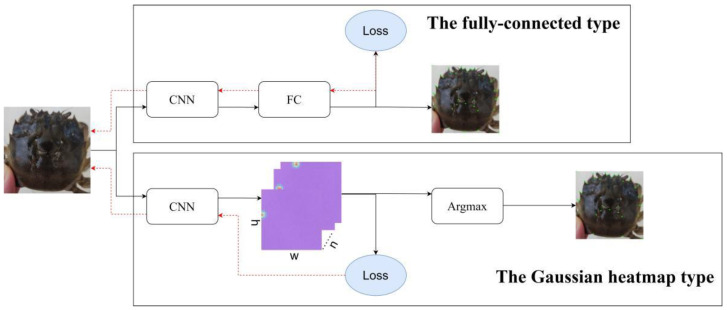
Schematic diagram of two typical feature point detection methods. Notes: The loss modules shown in the figure have various implementations depending on the experimental group. Nonetheless, all of these implementations aim to measure the loss between the predicted landmark coordinates and their corresponding actual coordinates. In this figure, the CNN module refers to the convolutional neural network, the FC module represents the fully connected layer, and the Argmax module corresponds to the Argmax function. In the Gaussian heatmap method, the symbol “*n*” denotes the number of feature points, “w” refers to the image width, and “h” represents the image height.

Regarding the network design methods, it is noted that, in the field of the Chinese mitten crab, manual annotation is the primary method for landmark point detection, while, in face recognition, landmark detection has been extensively studied. Therefore, there is a relatively complete methodology for landmark point detection. There are two main types of landmark detection methods based on the way coordinate points are obtained: fully connected and Gaussian heatmap. Both methods use a convolutional neural network to extract image features. The fully connected method uses a fully connected layer to map the feature map to the coordinate points. In the Gaussian heatmap method, the network outputs a feature map with 2*n* channels, where *n* represents the number of landmark points to be detected (in this study, *n* = 39, corresponding to the 37 carapace points plus the upper-left and lower-right corners of the bounding rectangle, as detailed in Section 2.2.1). Each pair of channels corresponds to the x and y coordinates of a single landmark point, reflecting the possible distribution of these coordinates in the heatmap. The loss module calculates the loss value between the predicted coordinates and the actual landmark point coordinates, and the CNN module represents the convolutional neural network, the FC module represents the fully connected layer, and the Argmax module represents the Argmax function. In the Gaussian heatmap method, *n* represents the number of feature points, and w and h represent the image’s width and height, respectively. These two landmark point detection methods are shown in Figure 3.

##### The Fully Connected Method

The fully connected method is the most straightforward approach for extracting coordinate points from feature maps. While the fully connected method offers advantages such as end-to-end differentiability and rapid training convergence, its limitations are equally notable. However, its spatial generalization ability is relatively poor as it focuses on global features. After sufficient training, each feature point will form a relatively fixed relative relationship. Due to spatially shared parameters, the convolutional neural network has a certain generalization ability. In the field of facial feature point recognition, the model’s generalization ability and training speed are usually further improved by optimizing the convolutional neural network and loss function.

MobileFaceNet [21] is a prominent face verification model that is tailored for high-accuracy real-time face verification in mobile and embedded devices. Compared to MobileNetV2, MobileFaceNet achieves more than two times the inference speed and better accuracy. Its 4MB model achieves 99.55% and 92.59% accuracy on LFW and MegaFace [22], respectively. Structurally, the main difference between MobileFaceNet and MobileNetV2 is that the global average pooling is optimized into a global depthwise convolution (GDConv). Inspired by this optimization method, the current study uses GDConv to replace the traditional global average pooling when designing the primary convolutional neural network, adding learnable weights for each position. The calculation method of GDConv is shown in Equation (1), where the summation is performed over the spatial dimensions of the input feature map.

(1) $$G_{1,1,k}=\sum_{i=1}^{w}\sum_{j=1}^{h}F_{i,j,k}\times K_{i,j,k}$$

**Figure 4:**
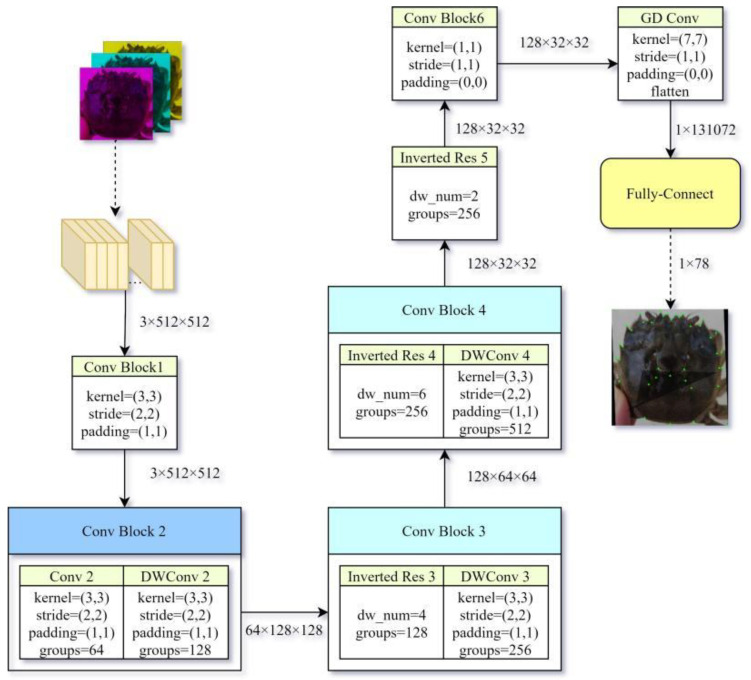
The schematic diagram of network forward transfer process in the fully connect method. Notes: In the figure, the size of the feature vector after passing through the previous modules is marked after the arrow. Some critical parameters and the sub-blocks it contains are marked in each block. DW Conv in the figure represents the depthwise convolution operation. The Inverted Res module represents the inverted residual module, and the parameter dw_num in the Inverted Res module represents the number of depth-wise convolution operations in the inverted residual module. The parameter groups in the Conv module represents the number of groups when grouping convolutions. After the fully connected layer, the feature vector is 1 × 78. In addition to the 37 landmark points, the upper-left and lower-right corners of the rectangular frame containing the Chinese mitten crab are also regarded as two feature points. The two-dimensional coordinates of the 39 points include a total of 78 numbers.

In Formula (1), *F* is the input feature map of size w×h×m, K is the convolution kernel of the same size w×h×m, and G is the output of size 1×1×m. The indices i and j represent the row and column positions in the spatial dimensions (ranging from 1 to w and 1 to h, respectively), while k denotes the channel index (ranging from 1 to m). In the design of the network in the current study, the grouped convolution and inverted residual modules are also used to reduce the amount of computation and perform fast downsampling. The overall network design is shown in Figure 4.

##### The Gaussian Heatmap Method

**Figure 5:**
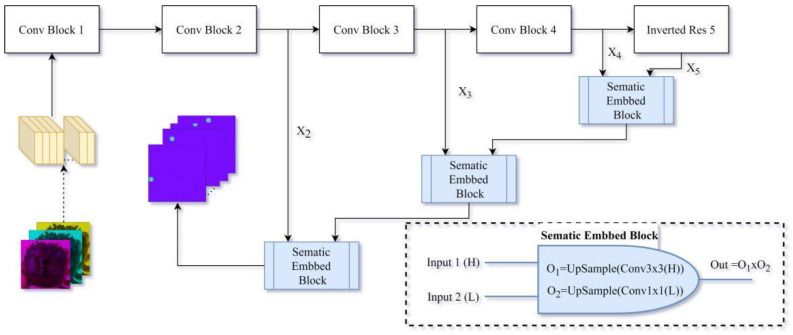
The schematic diagram of network forward transfer process in the Gaussian heatmap method. Notes: The structures of modules such as Conv Block 1, Conv Block 2, Conv Block 3, Conv Block 4, and Inverted Res 5 in Figure 5 are the same as the corresponding modules in Figure 4. The lower-right corner shows the specific structure of the Semantical Embedding Block (SEB). The Up Sample method used in SEB is the nn. UpsamplingBilinear2d method provided by PyTorch.nn. The thermal circles depicted here represent the predictive outcomes for each landmark point, with each channel corresponding to an individual landmark point.

In addition to designing a fully connected network based on the MobileNetV2 network skeleton, a convolutional neural network using the Gaussian heatmap method is also designed. The forward pass process of the convolutional neural network in the Gaussian heatmap method is shown in Figure 5. The Gaussian heatmap method uses grouped convolution, inverted residual structure, and other techniques to downsample features. Unlike the fully connected convolutional neural network, the Gaussian heatmap method uses the Semantical Embedding Block (SEB) for upsampling. The SEB structure is inspired by the classic YOLOV3 network [23], and its structure is marked in the lower-right corner of Figure 5. The input of each SEB includes two feature matrices of high and low dimensions. The SEB module performs a convolution operation with a 3 × 3 convolution kernel on the high-dimensional feature matrix and then upsamples it. The convolution kernel for the low-dimensional feature matrix is 1 × 1, and then it is also upsampled. Finally, the processed high-dimensional matrix is multiplied by the processed low-dimensional matrix to obtain the output of the SEB module. The entire forward pass process goes through a total of three SEB modules, retaining the characteristics of each stage. In the Gaussian heatmap mode, the network’s output is a 39 × 512 × 512 matrix, representing 39 heatmaps of 512 × 512 pixels, each of which reflects the location of a landmark point.

##### The Method of Differentiable Space-Numerical Transformation

The current study designs a differentiable implementation suitable for detecting landmark points of the Chinese mitten crab carapace based on the Differentiable Spatial to Numerical Transform [17] (DSNT) structure to overcome the shortcomings of the previous two methods. The method of differentiable space-numerical transformation is a novel approach designed to detect landmark points of the Chinese mitten crab carapace. It is based on the Differentiable Spatial to Numerical Transform (DSNT) structure, which has good spatial generalization ability while being fully differentiable.

The Differentiable Spatial to Numerical Transform (DSNT) method is designed to detect landmark points on the Chinese mitten crab carapace with strong spatial generalization and full differentiability. The Algorithm 1 provides the mathematical specification of the differentiable space-numerical transformation. The image is first converted into a 3 × 512 × 512 matrix. A feature matrix of size 128 × 32 × 32 is obtained through a fully convolutional neural network (as in Figure 4, excluding the fully connected layer). A subsequent convolution yields a 39 × 32 × 32 matrix, where 39 denotes the number of landmark points. This matrix is input into the DSNT module, producing an output heatmap Z, which is normalized per channel using the Softmax function to obtain Z′. Two coordinate matrices, X and Y, are defined with the same dimensions as Z (32 × 32), with elements standardized between −1 and 1. Specifically, $X_{i,j}=2\left(j-1\right)/\left(n-1\right)-1$ and $Y_{i,j}=2\left(i-1\right)/\left(m-1\right)-1$, where i and j are row and column indices, m=32 (height), and n=32 (width), ensuring unique values per position. This design, where $X_{i,j}$ is constant across rows and $Y_{i,j}$ across columns, is intentional: X and Y serve as one-dimensional reference axes, while the full two-dimensional spatial information is encoded in $Z_{k}^{\prime }$. The expected coordinates for each channel are computed as the scalar product of Z′ with X and Y, respectively: $x_{k}=<Z_{k}^{\prime },X>$ and $y_{k}=<Z_{k}^{\prime },Y>$, where <A, B> denotes the element-wise product summed over all elements (equivalent to the Frobenius inner product). The loss is the mean of the Euclidean loss (mean squared error, MSE) between predicted and actual coordinates and a regularization term on the heatmap distribution. Convergence is defined as the stabilization of this loss below a threshold (0.001) over 300 epochs, as observed in Section 3.1.
**Algorithm 1**. Differentiable space-numerical transformationWhile loss > 0.001:  Through the fully convolutional neural network  Through the DSNT module  For each channel k:   Normalize $Z_{k}$ using Softmax to get $Z_{k}^{\prime }:\;$   $Z_{k,i,j}^{\prime }=\frac{e^{Z_{k,i,j}}}{\sum_{p=1}^{m}\sum_{q=1}^{n}e^{Z_{k,p,q}}}$, i=1,2,…,m; j=1, 2,…,n (with m=32,n=32)   Define $X_{i,j}=\frac{2j-(n+1)}{n}$, *i* = 1,2, ……, *m*; *j* = 1, 2, ……, *n*   Define $Y_{i,j}=\frac{2i-(m+1)}{m}$, *i* = 1, 2, ……, *m*; *j* = 1, 2, ……, *n*
   Compute $x_{k}=<Z_{k}^{\prime },X>$   Compute $y_{k}=<Z_{k}^{\prime },Y>$
  Calculate loss as mean of Euclidean Loss (MSE) and regularization loss  Update model parameters

Notes: The fully convolutional neural network in the above pseudo code is the same as the first half of the CNN in Figure 4, except that it does not go through the fully connected layer. m, *n* represent the width and height of the feature matrix, respectively. ${<A,\;B>}_{F}$ represents the result of multiplying and adding the corresponding elements in the matrix A and B. The calculated loss is the mean of the Euclidean loss and the regression loss.

#### 2.2.3. Design of Parallel Experiments

The current study designed seven groups of parallel experiments to compare the advantages and disadvantages of various experimental methods. These experiments were conducted on a server equipped with an NVIDIA GV100GL GPU (NVIDIA Corporation, Santa Clara, CA, USA), running Ubuntu 20.04.2 OS, and utilized Python 3.8, PyTorch 1.9.0, and CUDA 11.1. The parallel experiment design compared the advantages of different schemes from the perspectives of loss function, data, and network structure. The loss function in these experiments adopted traditional L1 loss, Smooth L1 loss [24], and Wing loss [25]. The dataset used was the source crab carapace dataset and the augmented dataset. Additionally, the neural network adopted the three network structures described in Section 2.2.2. The specific experimental design is shown in Table 1. Groups 1–3 were used to compare the three loss functions, while Groups 1–3 and 4–7 were used to compare the role of data augmentation. Groups 6 and 7 explored the effect of Gaussian heatmap and differentiable space-numerical transformation neural network in landmark points recognition of Chinese mitten crab carapace.

## 3. Results

### 3.1. Training Results

**Figure 6:**
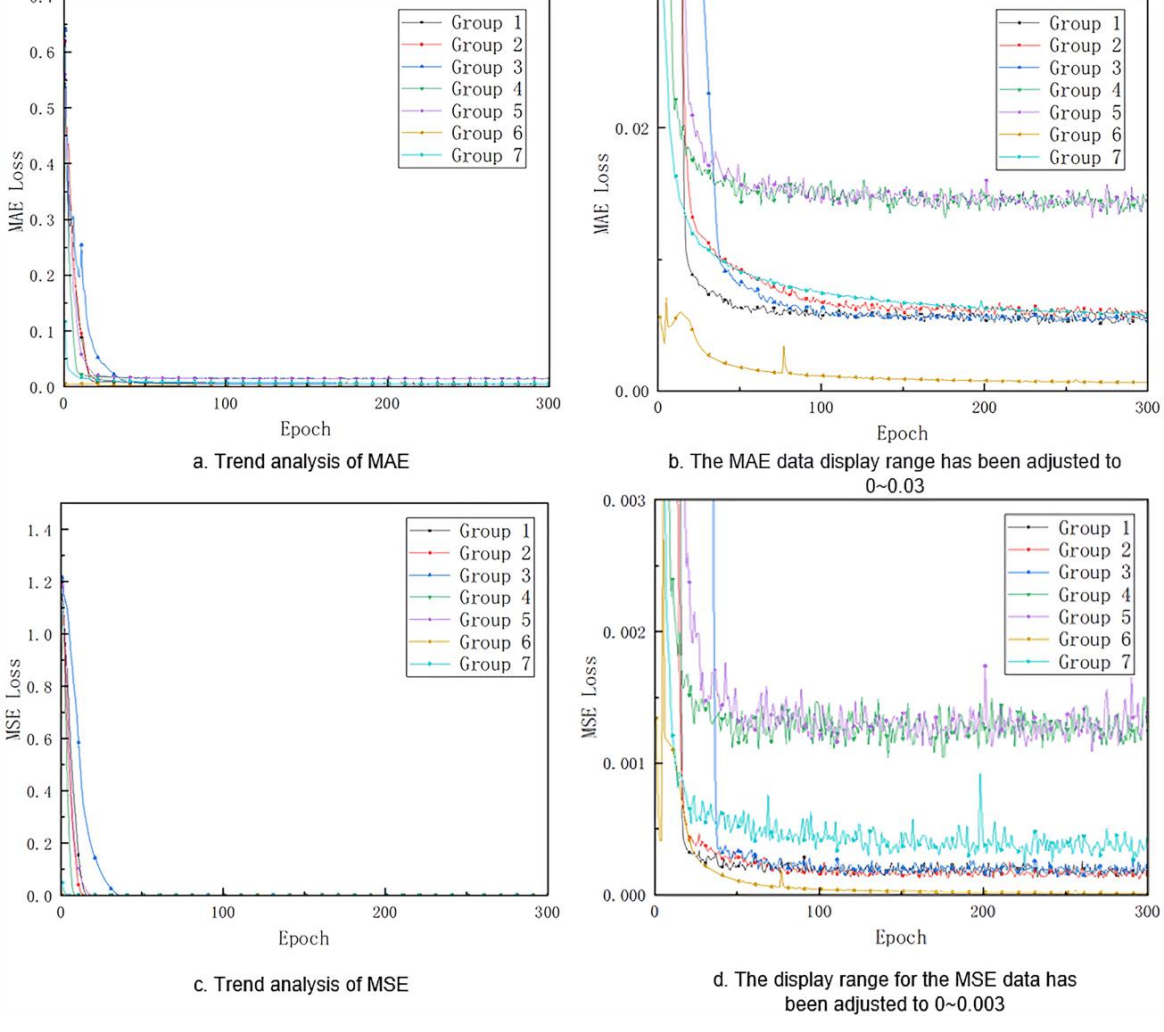
The changing process of MAE loss and MSE loss in the 300 epochs of training. Notes: The group design in this figure is the same as in Table 1.

The experimental results indicate that the choice of loss function significantly affects the model’s recognition performance. Figure 6 shows the changes in the Mean Absolute Error (MAE) and Mean Squared Error (MSE) loss values for each group during the 300 epochs of training. Specifically, Figure 6a,b shows the changes in MAE loss, while Figure 6c,d shows the changes in MSE loss. As shown in Figure 6a,c, both MAE and MSE loss values for each group converge within 0.1 after 50 epochs of training. Therefore, it is difficult to observe significant changes in the loss values after the 50th epoch in Figure 6a,c. To better observe the differences between the groups, the display area for Figure 6a,c is reduced to 0–0.03 and 0–0.003, respectively, in Figure 6b,d. Additionally, since Groups 1–3 use the original dataset while Groups 4–7 use the augmented dataset, direct comparison of the MAE and MSE loss values between these two large groups is not straightforward.

The results of the training process indicate that the choice of loss function significantly affects the model’s recognition performance. As shown in Figure 6, Both MAE and MSE loss values across all experimental groups converged below 0.1 within the initial 50 training epochs, making it difficult to observe the parameter changes after this point. However, by reducing the display area for MAE and MSE loss in Figure 6b,d, the performance differences between each group become more apparent. For example, among the three groups in Groups 1–3, Group 1 exhibits the fastest convergence, while Group 3 converges slightly faster than Group 2. In terms of the augmented datasets used by Groups 4–7, Group 4 shows a slightly faster convergence rate than Group 5, with both groups achieving similar final loss values. Based on the training process results, traditional L1 loss outperforms Smooth L1 loss and Wing loss. Furthermore, Wing loss shows slightly better performance than Smooth L1 loss. When comparing the different network structures, Gaussian heatmap-based CNN exhibits the best performance, followed by DSNT-based CNN, while fully connect-based CNN performs the worst. Group 6 achieves convergence within the minimum number of epochs, with the smallest convergence value, followed by Group 7, and finally Group 4, which achieves convergence with the maximum number of epochs and the largest convergence value.

**Figure 7:**
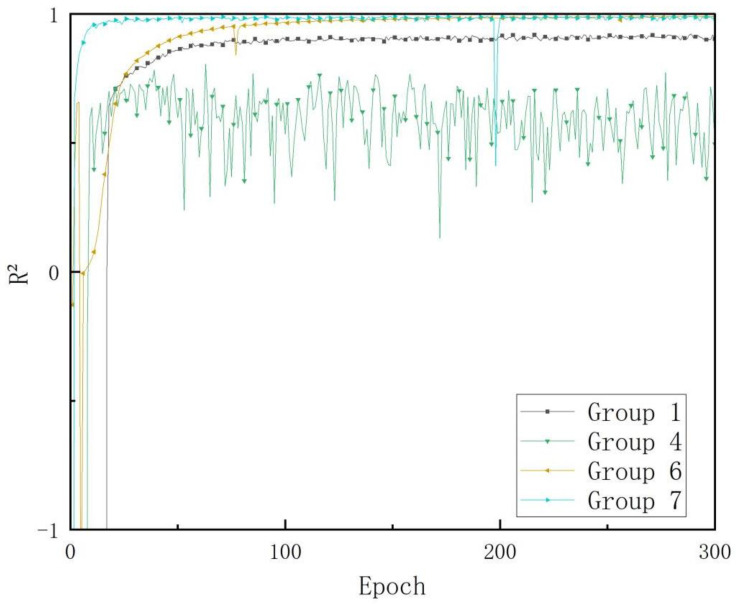
The changing process of R squared in the 300 epochs of training. Notes: The group design in this figure is the same as in Table 1.

In addition to the loss value, R^2^ is also a common regression evaluation criterion. R^2^ (R squared, coefficient of determination) reflects the accuracy of the model in fitting the data. Generally, R^2^ values range from 0 to 1, with values closer to 1 indicating a stronger explanatory power of the independent variable in the model equation to the dependent variable, and a better fit of the model to the data. Analysis of the MAE and MSE loss of the seven experimental groups shows that Group 1 outperforms the first three experimental groups. In the two groups of Groups 4 and 5, Group 4 performs slightly better, and Group 6 and 7 have similar performances (with Group 6 being slightly better). The change process of R^2^ for the four groups of trials in Group 1, Group 4, Group 6, and Group 7 during the 300 epochs of training is shown in Figure 7.

It is worth noting that R^2^ is a commonly used regression evaluation metric that reflects the accuracy of model fitting. As shown in Figure 7, the R^2^ values for the four groups of experiments from Group 1, 4, 6, and 7 are displayed during the 300 epochs of training. The results indicate that Group 4 has a significantly lower R^2^ performance than the other three groups. Notably, Group 4 employs an augmented dataset, L1 loss, and a fully connected convolutional neural network, and the inferior performance of this group highlights the weak generalization ability of the fully connected method in landmark detection. Despite the training data already containing distorted and deformed samples, the violent fluctuations in the graph demonstrate the unsatisfactory spatial generalization capability of the fully connected method. In contrast, the R^2^ performance of Groups 6 and 7 is superior to that of Group 1. Group 7 attains convergence at a slightly lower epoch number than Group 6, but the R^2^ values for both sets of experiments are similar. Therefore, it is necessary to comprehensively evaluate the advantages and disadvantages of Groups 6 and 7 from other perspectives, such as model size and computational cost.

### 3.2. Results in the Test Set

Results obtained in the training set may be influenced by factors such as overfitting, thus the untrained test set provides a better reflection of the model’s generalization and actual detection ability. The R^2^ values of the seven groups of models on the test set are presented in Table 2, which was used as the evaluation criterion.

From Table 2, it is evident that the first three groups performed significantly worse than the last four. One possible explanation is that the training data set used in the first three groups did not include augmented data such as distortion and deformation, which was present in the test set. Of all the groups, Group 7 achieved the highest predictive accuracy, with an R^2^ value of 0.9906 on the test set. Among the first three sets of experiments, the model from Group 1 showed the best performance. Group 4 and Group 5 had similar performance, with Group 5 slightly outperforming Group 4.

**Figure 8:**
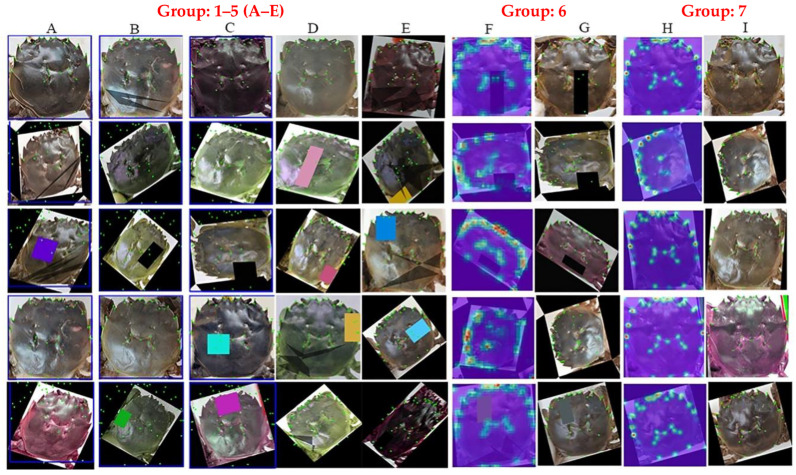
Partial visualization of results for each group of models. Notes: Columns A–E are the model visualization results for Groups 1–5. Columns F and G are the thermal distribution results and the landmark-label results of the Group 6 model, respectively. Column H and Column I are the thermal distribution results and the landmark-label results of the Group 7 model, respectively.

In addition to differences in parameters such as R^2^, there were also noticeable differences in the actual effect of landmark recognition in each group. To visually compare the ability of each group of models to identify the landmark points of Chinese mitten crab carapaces, five images of crab carapaces were randomly selected from each group of models, as shown in Figure 8.

**Figure 9:**
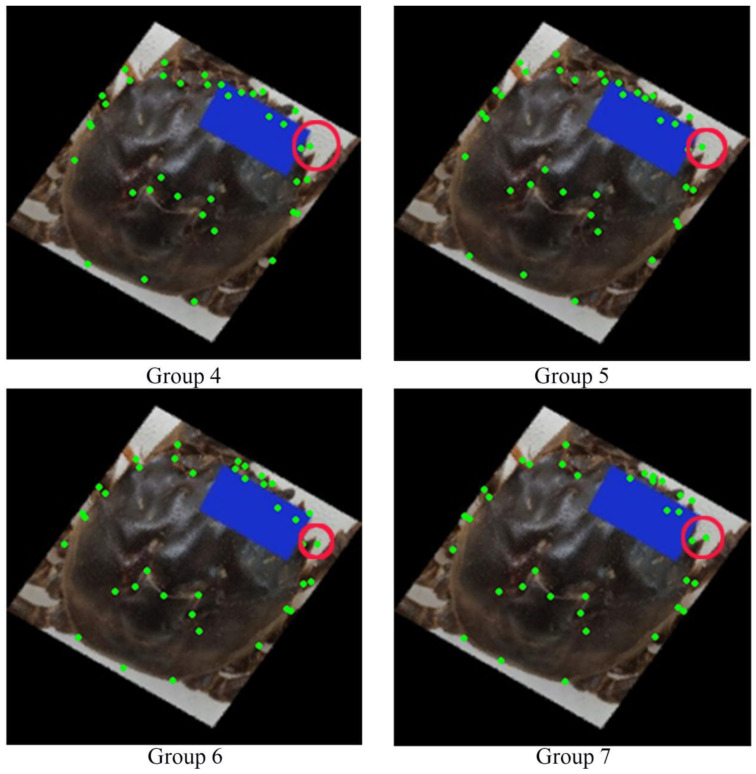
Comparison of detail extraction effects of Groups 4–7 pairs of deformed sample landmarks.

Figure 8 presents a clear visualization of the ability of each group of models to identify the landmark points of the Chinese mitten crab carapace. Among the seven groups of samples, Groups 6 and 7 (columns F–I in Figure 8) demonstrate the most accurate identification, and the positions of the identified points are consistent with human eye observations. Models trained by Groups 1–3 (columns A–C in Figure 8) have good recognition ability for samples with correct angles but poor recognition ability for samples that have been rotated or otherwise deformed. Groups 4 and 5 (column D and column E in Figure 8) significantly improved the ability to recognize deformed sample contours. However, there is still a significant deviation in the details of the feature points, which are not easily observed in Figure 8. Therefore, Figure 9 uses Groups 4–7 to identify the same deformed sample, making the difference more obvious.

In Figure 9, the outlines of the crab carapaces in Groups 4 and 5 are consistent, but there are many problems in detail. One undeniable example is the M-shaped neck groove. The M-shaped neck grooves identified in Groups 4 and 5 are higher than the correct samples, while the M-shaped neck grooves identified in Groups 6 and 7 are very accurate. From the sample analysis in Figure 9 alone, the detail extraction effect of Group 7 is better than that of Group 6. For example, the second right tooth (P22 in Figure 1c) identified by the Group 7 model for this sample falls precisely on the tooth peak, while the Group 6 model has a biased identification of this point.

### 3.3. Model Power Consumption Related Results

In addition to evaluating the accuracy of CNN models, data related to power consumption, such as training speed, number of parameters, amount of calculation, and model size, are also important criteria for assessing the models. As production equipment conditions are often limited, models with fast training speed, small parameters, and a small amount of calculation are more applicable in real-world scenarios. Table 3 presents the power consumption-related data of each group of models in the current study.

According to Table 3, the training time of the source dataset (Groups 1–3) is lower than that of the augmented dataset. Among the augmented dataset, the DSNT-based CNN model’s training time is lower than that of the fully connected CNN, let alone the Gaussian heatmap-based CNN. The training time based on the Gaussian heatmap-based CNN has reached 224.01 h, 26.89 times that of the DSNT-based CNN (8.33 h). The network structures of Groups 1–5 are the same, with differences in datasets and loss functions only, resulting in identical forward inference parameters, floating-point operations, and model sizes. However, due to the use of the fully connected layer, the forward inference parameters, floating-point operations, and model sizes of the five groups of models are the largest among these groups (the forward inference parameters reached 27.93 M, the floating-point operations reached 159.58 G, and the model size reached 106.37 MB). The DSNT-based CNN model’s parameters, computation, and model size are smaller than those of the Gaussian heatmap-based model. Therefore, based on the above analysis, the CNN model based on DSNT performs best in recognizing the landmark points of the cephalothorax carapace of the Chinese mitten crab.

## 4. Discussion

The current study aimed to explore the efficiency and accuracy of three different CNNs for detecting landmark points on the carapace of Chinese mitten crabs. The results indicated that the DSNT-based CNN model was the most suitable, as it achieved high accuracy, was efficient, and lightweight. In contrast, the fully connected CNN and Gaussian heatmap-based CNN models, which have been extensively applied in facial landmark point location and recognition [26,27] and emerging fields such as intelligent driving [28], had their limitations. Previous studies on these models for human face and skeleton landmark detection had identified their respective weaknesses, which were caused by the limited space for local filter weights in CNNs. Consequently, CNNs could not always locate the part of the image that they were looking at. Landmark point detection requires location information, which CNNs could not always provide implicitly, despite experimental evidence to the contrary. Islam et al. [29] hypothesized that deep CNNs could learn this location information implicitly and have been experimentally verified. However, the experimental results in the current study show that this level of implicit learning is not enough to achieve the accuracy of landmark point detection. The bottom-up parsing method proposed by Cao et al. [30] achieves real-time performance while ensuring high accuracy. This bottom-up approach generates a heatmap for each feature point as a channel. This CNN detection method based on Gaussian heatmap is better than the traditional fully connected layer CNN model as a whole, which has also been verified in the experiments of the current study. Due to the presence of Argmax, there is a lower error bound for the Gaussian heatmap-based CNN model. For high-resolution images, these lower bounds of error are negligible. Nevertheless, for low-resolution images, this lower error limit can be fatal. In addition, the CNN model based on the Gaussian heatmap still has the problems of slow training speed and large memory consumption. The superior performance of the DSNT-based CNN model can be theoretically attributed to its integration of spatial generalization and end-to-end differentiability. Unlike the fully connected method, which relies heavily on global feature mapping and thus struggles with spatial variability (Section 2.2.2), DSNT leverages a heatmap-to-coordinate transformation that preserves local spatial relationships while allowing gradient flow through the entire network. This is achieved by normalizing the heatmap with Softmax and computing coordinate expectations via the Frobenius norm, enabling precise localization without the memory-intensive intermediate representations required by Gaussian heatmaps (Section 2.2.2). Compared to the Gaussian heatmap method, which introduces discretization errors due to the Argmax operation, DSNT’s differentiable nature eliminates such errors, enhancing accuracy especially at lower resolutions (Section 4). This theoretical foundation explains the observed R^2^ value of 0.9906 on the test set (Section 3.2) and the reduced computational footprint (Section 3.3), positioning DSNT as an optimal balance between precision and efficiency. In conclusion, the DSNT-based CNN model demonstrated superior performance in carapace landmark detection for Chinese mitten crabs, achieving an optimal balance between precision and computational efficiency.

The best landmark point regression method in the current study achieved an R^2^ value of 0.9906 for 37 landmark points. High-precision landmark point detection has the potential to revolutionize various aspects of the Chinese mitten crab industry. Currently, most processes related to breeding, observation, and processing of Chinese mitten crabs rely heavily on manual labor, which limits the progress of the industry. For instance, trait measurements of crabs require labor-intensive manual measurements [31], which are further complicated by the crab’s intense vitality. Precise landmark point detection provides an effective solution to this problem. By placing a reference object at the same vertical height as the crab, the length and width of the carapace can be accurately measured based on the number of pixels. Moreover, Chinese mitten crabs in different watersheds exhibit significant differences in taste, quality, and price. Some studies have attempted to differentiate Chinese mitten crabs from different watersheds based on their morphology, with promising results. Zheng et al. [32] employed landmark point detection to quantify the shape characteristics of crabs from eight different watersheds in China. Zheng observed differences in the shape of the carapace and abdomen of Chinese mitten crabs from different watersheds using manual labeling and principal component analysis. However, his study did not mention landmark point detection accuracy and regression accuracy and hence cannot be compared to the results of the current study. In the initial stages of the current research, the algorithm also considered the use of principal component analysis. However, the regression effect of this method was far inferior to that of various CNN methods. Therefore, applying the DSNT-based CNN model from the current study to Zheng’s work may yield more convincing conclusions.

In real-world aquaculture settings, the applicability of the model is influenced by various external factors, such as lighting conditions, camera equipment, and image resolution variations. The data collection in this study was performed under natural lighting conditions using a OnePlus 6T smartphone, with relatively controlled illumination (9:00–18:00). However, actual aquaculture environments may involve low-light conditions, water surface reflections, or nighttime operations. To assess the model’s robustness, the superior generalization ability of the DSNT-based CNN may enable it to perform better under diverse lighting conditions compared to the fully connected method, which exhibits weaker adaptability outside the training data distribution (see Section 3.2). Furthermore, differences in camera equipment resolution and quality could impact landmark detection accuracy. The DSNT approach, with its end-to-end coordinate regression, reduces the dependency on high resolution seen in the Gaussian heatmap method (Section 4), suggesting potential resilience even with lower-resolution devices. Future work should validate the model under real-world aquaculture conditions, incorporating diverse camera resolutions and challenging illumination scenarios to further validate its practical utility.

While the DSNT-based CNN model achieved an R^2^ value of 0.9906 on the test set (Section 3.2), there remains a possibility of overfitting, particularly given the controlled nature of the training and test datasets. Data augmentation with random distortions, rotations, and occlusions (Section 2.2.1) improved generalization, as evidenced by the performance gap between Groups 1–3 (no augmentation) and Groups 4–7 (augmented data) in Table 2. However, these augmentations may not fully capture the complexity of real-world aquaculture environments, such as variable water currents or biological fouling on crab carapaces. The current validation, limited to a test set derived from the same collection event (Section 2.1), lacks diversity in environmental conditions. To mitigate overfitting concerns and ensure robustness, future work should include cross-validation with datasets from multiple aquaculture sites and real-time testing in operational settings, complementing the controlled experimental results presented here. This study compared three CNN-based approaches but did not include traditional computer vision techniques such as Harris corner detection or SIFT, which have been foundational in landmark detection (Section 1). These methods excel in detecting well-defined corners or keypoints in structured scenes but struggle with the irregular textures and subtle morphological variations of Chinese mitten crab carapaces, as noted in prior ecological studies. The decision to focus on CNNs was driven by their ability to learn hierarchical features directly from raw images, offering greater flexibility for this task. However, a comparative baseline with traditional methods could provide additional context for the DSNT-based CNN’s performance. Future research could incorporate such comparisons to quantify the trade-offs between computational complexity and detection accuracy in this domain.

Due to various limitations, such as time and resources, the present study did not explore the practical applications of accurately detecting the landmark points of the Chinese mitten crab. However, the results of this study can be utilized in future research to conduct studies on the quality evaluation and traceability of Chinese mitten crabs. By combining accurate landmark point detection with other data, such as water quality, breeding environment, and food sources, the quality and origin of Chinese mitten crabs can be more precisely identified and evaluated. In addition, the detection of landmark points can be integrated into automated processing systems, which can improve the efficiency and accuracy of crab processing. Further studies could investigate the application of landmark point detection in other crustaceans, such as integrating it with growth-related models of other crab and shrimp species for age determination [33]. There may be analogous research needs in these species.

## 5. Conclusions

In conclusion, the current study demonstrates the effectiveness of using CNNs for landmark point detection in the carapace of the Chinese mitten crab. Through comparison and analysis, it was found that the DSNT-based CNN model outperformed the other models in terms of accuracy and efficiency. The high-precision landmark point detection achieved in this study has great potential for applications in various aspects of the Chinese mitten crab industry, such as breeding, observation, processing, and quality evaluation. Future research can utilize these results to further explore the potential of landmark point detection in the Chinese mitten crab industry. Overall, this study provides a valuable contribution to the field of computer vision and its application in aquatic animal research.

## Figures and Tables

**Table 1:** Design of parallel experiments.

Groups	Loss Functions				Data Sets		Networks		
	L1	Smooth	Wing	Others	Source	Augmented	FC	HM	DSNT
Group 1	√				√		√		
Group 2		√			√		√		
Group 3			√		√		√		
Group 4	√					√	√		
Group 5	√					√	√		
Group 6	√					√		√	
Group 7				√		√			√

Notes: In the Loss Functions column, L1 represents L1 loss, Smooth represents Smooth L1 loss, Wing represents Wing loss, and Others represents loss functions other than these three types. It is important to note that only Group 7 used a combined regression loss and Euclidean loss combined loss function. In the data sets column, “Source” represents the original crab carapace dataset, and “Augmented” represents the augmented dataset. In the networks column, “FC” represents a convolutional neural network whose last layer is a fully connected layer, “HM” represents a Gaussian heatmap neural network, and “DSNT” represents a convolutional neural network based on differentiable space-numerical transformation. The results of these experiments are presented and analyzed in Section 3.

**Table 2:** The R^2^ value of each group of models on the test set.

Groups	R^2^ Value on the Test Set
Group 1	0.8755
Group 2	0.8451
Group 3	0.8690
Group 4	0.9830
Group 5	0.9833
Group 6	0.9846
Group 7	0.9906

Notes: The group design in this table is the same as in Table 1. The number of test set samples is one-tenth of the full augmented data set.

**Table 3:** Comparison of resource usage for each group of models.

Groups	Training Time (h)	Parameters (M)	FLOPs (G)	Model Size (MB)
Group 1	5.53	27.93	159.58	106.37
Group 2	7.06	27.93	159.58	106.37
Group 3	5.54	27.93	159.58	106.37
Group 4	8.57	27.93	159.58	106.37
Group 5	8.59	27.93	159.58	106.37
Group 6	224.01	0.97	156.05	4.23
Group 7	8.33	0.84	88.34	3.67

Notes: The Training time value represents the time consumed by 300 epochs, the Parameters value represents the number of parameters in forwarding inference for each group of models, and the FLOPs (floating point operations) value represents the number of floating point operations performed during forwarding inference.

## Data Availability

The original contributions presented in the study are included in the article; further inquiries can be directed to the corresponding author.
